# Choroidal Thickness and Urinary Albumin Excretion in Type 2 Diabetic Patients without Retinopathy

**DOI:** 10.1155/2020/3648941

**Published:** 2020-02-25

**Authors:** Cláudia Oliveira-Ferreira, Mariana Leuzinger-Dias, João Tavares-Ferreira, F. Falcão-Reis, Amândio Rocha-Sousa

**Affiliations:** ^1^Ophthalmology Department, Centro Hospitalar São João, Porto, Portugal; ^2^Department of Surgery and Physiology, Faculty of Medicine of Porto University, Porto, Portugal

## Abstract

The role of retinal vasculature's dysfunction in the physiopathology of Diabetic Retinopathy (DR) has been extensively described. Recently, the existence of a diabetic choroidal vasculopathy has been proposed. The purpose of this study was to compare choroidal thickness (CT) in nondiabetic patients and in type 2 diabetic patients without retinopathy, using EDI SD-OCT. Additionally, considering the diabetic patient group, compare CT in patients with and without microalbuminuria. This retrospective study selected patients sent from primary health-care centers as part of the national screening of diabetic retinopathy. Inclusion criteria were diagnosis of type 2 diabetes mellitus, absence of diabetic retinopathy, and a 24 hours urinary albumin measurement in the last 3 months at the primary health-care center. Nondiabetic patients were selected from a database in the ophthalmology department, and only healthy patients were included. At the screening visit, all patients performed a complete ophthalmologic examination by the same examiner. All eyes were examined with SD- OCT, and all scans were performed in the EDI mode. Measurements were made at three points: subfoveal, 1500 *μ*m temporally and nasally to the foveal center. We included 110 eyes of 110 diabetic patients without diabetic retinopathy and 30 eyes of 30 healthy controls. Mean subfoveal CT was greater in diabetic patients without retinopathy (with normoalbuminuria or microalbuminuria) when compared with nondiabetic patients (*p* < 0.05). In diabetic patients without retinopathy, the subfoveal and temporal choroid was thicker among patients with microalbuminuria when compared with those of normoalbuminuric patients (*p* < 0.05). The subfoveal and temporal choroid was thicker among diabetic patients with microalbuminuria compared with nondiabetic patients. (*p* < 0.05). This study suggests that choroidal changes are present in type 2 diabetic patients even before the clinical development of retinopathy.

## 1. Introduction

Diabetes mellitus is one of the greatest global health issues of the 21^st^ century, representing a huge burden to today's health-care systems [1]. Around 415 million people worldwide are estimated to live with the disease, with this number expected to increase to 642 million by 2040 [1]. Diabetic retinopathy (DR) is the leading cause of visual loss in working-age adults, affecting more than 35% of diabetic patients [2].

The role of retinal vasculature's dysfunction in the physiopathology of DR has been extensively described. However, much less attention has been given to choroidal vessels, thus far [3]. Recently, though, the existence of a diabetic choroidal vasculopathy has been proposed [4–8]. This diabetic choroidopathy (DC), as it has been called, includes increased vascular tortuosity, microaneurysms, obstruction of the choriocapillaris, nonperfusion areas, and choroidal neovascularization [4].

In fact, the choroid is the most highly vascularized structure in the body, receiving 95% of all ocular blood flow. It is responsible for the blood supply of the outer retina and avascular fovea, maintaining the extremely metabolically active photoreceptor cells [9]. Consequently, choroidal hypoperfusion could result in outer retina's dysfunction [5, 10]. Joining all these pieces together, it has been theorized that DC may be responsible for unexplained vision loss in diabetic patients without DR [5].

Microalbuminuria is an early marker of generalized endothelial damage, and it is associated with microvascular chronic complications in diabetic patients. Annually, 5%–10% of type 2 diabetes mellitus patients with microalbuminuria develop diabetic nephropathy, presenting an increased risk of developing DR [11, 12].

For a long time, choroidal observation and evaluation have been limited by its deep location, behind the retinal pigment epithelium. The emergence of spectral-domain (SD) optical coherence tomography (OCT) systems and the enhanced depth imaging (EDI) mode has led to a more efficient observation and measurement of the choroid [12].

The purpose of this study was to compare choroidal thickness (CT) in nondiabetic patients and in type 2 diabetic patients without retinopathy, using EDI SD-OCT. Additionally, considering the diabetic patient group, we compared CT in patients with and without microalbuminuria.

## 2. Material and Methods

This retrospective study selected patients sent from primary health-care centers as part of the national screening of diabetic retinopathy.

Inclusion criteria were diagnosis of type 2 diabetes mellitus, absence of diabetic retinopathy, and a 24 hours urinary albumin measurement in the last 3 months at the primary health-care center.

Exclusion criteria were diabetic retinopathy, other retinal pathology (including hard drusen or signs of chronic systemic hypertension), uveitis, refractive error of 6 or more diopters (D), ocular hypertension and/or glaucoma, ocular procedures such as capsulotomy, laser focal, and panretinal photocoagulation, history of ocular surgery (including intravitreal injections and cataract surgery), history of ocular/orbital trauma, systemic hypertension, systemic lupus erythematosus, anemia, leukemia, diagnosed obstructive sleep apnea, neurodegenerative disease, smoking, body mass index ≥40, and macroalbuminuria or reduced media transparency that makes OCT unfeasible.

Nondiabetic patients were selected from a database in the ophthalmology department (from general ophthalmology consultation), and only healthy patients were included.

At the screening visit, all patients performed a systemic and ocular history and a complete ophthalmologic examination by the same examiner: visual acuity with Snellen charts (and refractive error correction), anterior segment observation, tonometry (with Goldmann applanation), and fundoscopic examination with 90 D and 60 D lens.

All eyes were examined with SD-OCT (Spectralis Heidelberg Engineering, Germany), and all scans were performed in the EDI mode. CT was manually measured by the same experienced examiner, from the outer edge of the hyper‐reflective retinal pigment epithelium to the inner sclera. Measurements were made at three points: subfoveal, 1500 *μ*m temporally, and nasally to the foveal center.

Microalbuminuria was defined by urinary albumin in 24 hours between 30-300 mg/day. Urinary albumin <30 mg/day was considered normoalbuminuria and >300 mg/day was considered macroalbuminuria.

Data were analyzed using the Statistical Package for the Social Science for Windows, version 23.0 (IBM Corp. Released 2013. IBM SPSS Statistics for Windows, Version 22.0; IBM Corp, Armonk, NY). The categorical data were presented as percentages and the continuous variables as mean. Data normality was assessed using the Kolmogorov–Smirnov test of normality. Student's *t*-test was used to compare groups, and nonparametric chi-square and Mann–Whitney tests were used, as appropriate. *p* < 0.05 was considered statistically significant.

## 3. Results

We included 110 eyes of 110 diabetic patients without diabetic retinopathy (46 eyes in patients with normoalbuminuria and 64 eyes in patients with microalbuminuria) and 30 eyes of 30 healthy controls.

### 3.1. Diabetic Patients without Diabetic Retinopathy vs. Nondiabetic Patients

In the group of diabetic patients, 60.0% were male, the mean age was 66.67 ± 9.49 years (range 44–86), and the mean duration of diabetes diagnosis was 12.34 ± 8.46 years. There were no significant differences between the two groups.

In the group of diabetic patients, mean subfoveal CT was 251.08 ± 69.31 *μ*m (vs. 246.03 ± 59.41 *μ*m in nondiabetic patients). Nasal CT was 212.36 ± 70.99 *μ*m (vs. 210.33 ± 68.11 *μ*m in nondiabetic patients), and temporal CT was 243.91 ± 63.48 *μ*m (vs 241.87 ± 62.44 *μ*m in nondiabetic patients).

Mean subfoveal CT was greater in diabetic patients (with normoalbuminuria or microalbuminuria) when compared with nondiabetic patients (*p* < 0.05). No differences were observed in nasal and temporal CT between the groups (Table 1).

### 3.2. Diabetic Patients without Diabetic Retinopathy: Microalbuminuria vs. Normoalbuminuria

There were no differences regarding age, gender, and mean time of diabetes diagnosis between the groups.

The mean subfoveal CT in microalbuminuric patients was 252.53 ± 64.046 *μ*m (vs. 247.88 ± 81.144 *μ*m in normoalbuminuric), the mean nasal CT was 215.36 ± 65.552 *μ*m (vs. 205.75 ± 82.906 *μ*m in normoalbuminuric), and temporal CT was 245.09 ± 53.397 *μ*m (vs. 241.29 ± 82.822 *μ*m in normoalbuminuric).

The subfoveal and temporal choroid was thicker among patients with microalbuminuria compared with normoalbuminuric (*p* < 0.05). No differences were observed in nasal CT between the groups (Table 2).

### 3.3. Diabetic Patients without Diabetic Retinopathy and Microalbuminuria vs. Nondiabetic Patients

There were no differences regarding age, gender, and mean time of diabetes diagnosis between the groups.

The mean subfoveal CT in microalbuminuric patients was 252.53 ± 64.046 *μ*m (vs. 246.03 ± 59.41 *μ*m in the nondiabetic group), the mean nasal CT was 215.36 ± 65.552 *μ*m (vs. 210.33 ± 68.11 *μ*m in the nondiabetic group), and temporal CT was 245.09 ± 53.397 *μ*m (vs. 241.87 ± 62.44 *μ*m in the nondiabetic group).

The subfoveal and temporal choroid was thicker among patients with microalbuminuria compared with controls (*p* < 0.05). No differences were observed in nasal CT between the groups (Table 3).

## 4. Discussion and Conclusion

In this study, we analyzed the CT in the nondiabetic and diabetic groups. Both have a normal distribution of CT, with a thicker temporal than nasal quadrant, as showed in previous studies.

We find that the mean subfoveal choroid is thicker (with statistical significance) in diabetic patients without diabetic retinopathy when compared with nondiabetic patients. Previous studies, also using EDI SD- OCT, showed different and somehow contradictory results [13–17]. In some of them, CT seems to be thinner in diabetic patients without retinopathy (when compared with controls), while other studies report thicker choroids.

Vujosevic et al. observed that mean macular CT progressively and significantly decreased with increasing level of DR (nonproliferative and proliferative DR vs. controls), but no significant CT difference was found between controls and diabetic eyes without DR [13]. Esmaeelpour et al. observed that the central choroid is thinner in all type 2 diabetic eyes regardless of the disease stage (with and without DR) when compared with healthy patients [14]. Querques et al. found that, in diabetic eyes (with and without DR), there is an overall thinning of the choroid [18]. However, all these three studies presented a small sample regarding diabetic patients without DR (range 15–22).

On the other hand, Xu et al. demonstrated that patients with type 2 diabetes mellitus (with and without DR) had a slightly, but statistically significantly, thicker subfoveal choroid (nor the stage of diabetic retinopathy is significantly associated to CT increase) [15]. Tavares Ferreira et al. observed that CT was increased for diabetic patients without DR in all the locations evaluated (subfoveal choroid, at 500, 1000, 1500 from the fovea to nasal, temporal, inferior, and superior), but without statistical significance, except in a single location, 1,500 *μ*m superior of the fovea [19]. Both these studies had a bigger sample with 246 and 125 patients, respectively.

Flowmetry laser doppler studies showed that choriocapillaris is the most affected layer in early diabetic choroidopathy, while histological studies also show changes in the Sattler and Haller layers [3, 17, 20]. However, using the ocular pulsatile blood flow, the large choroidal vessels do not appear to be as affected as choriocapillaris [3].

The decrease in choroidal thickness can be explained by the loss of choroidal capillaries, increased choroidal vessels drop-out, increased vascular resistance, and decreased choroidal flow in the foveal region. These events may condition retinal hypoxia and increased VEGF levels resulting in the development of macular edema as a result of breakdown of the blood-retinal barrier [19, 21]. In support of this, studies with indocyanine green angiography show greater delays in choroidal vascular filling with increasing severity of DR [22]. Additionally, studies using laser Doppler flowmetry demonstrated that choroidal blood flow and volume are decreased in the foveal region in diabetic patients, even those without diabetic retinopathy [3, 7]. Thus, these studies suggest that the reduction in choroidal thickness in early diabetic choroidopathy might be associated with choriocapillaris changes that are too small to be detected.

On the other hand, some authors suggest that CT increase can be due to an increased production of VEGF or other cytokines mediating choroidal vasodilation, elevation in choroidal blood flow, which subsequently increase the thickness of the choroidal vascular layer or increase vascular permeability and consequent choroidal swelling [9, 15, 23].

We observed that subfoveal and temporal choroid was thicker among patients with microalbuminuria compared with normoalbuminuric (*p* < 0.05) and controls.

Farias et al. studied type 2 diabetes patients with no or mild DR and found a thinner subfoveal and temporal choroid CT in the microalbuminuric groups (when compared with normoalbuminuric) and a thinner subfoveal, temporal, and nasal CT in the microalbuminuric group when compared with controls [24]. Farias observed that mean subfoveal and temporal CT was significantly reduced in the microalbuminuric group when compared with normoalbuminuric [12].

Microalbuminuria corresponds to a state of generalized vascular dysfunction with consequent functional and structural abnormalities in the blood vessels, such as endothelial dysfunction and reduction of vascular compliance. CT reduction observed in diabetic patients without diabetic retinopathy and with microalbuminuria could be explained by this generalized dysfunction and can be interpreted as an early evidence of microvascular choroidal damage, even before the first signs of diabetic retinopathy, such as microaneurysms, can be detected [24].

Malerbi et al. studied type 1 DM and observed that, in diabetic patients without DR, the subfoveal, nasal, and temporal choroid was thicker (when compared with nondiabetic patients). They also observed that subfoveal choroid was thicker in microalbuminuric when compared with normoalbuminuric patients without DR [25]. The explanation to increased CT in diabetic patients without retinopathy (vs. controls) and in microalbuminuric patients (vs. normoalbuminuric and controls) in our and other studies, could be associated with increased vascular permeability and/or vasodilation, autonomic dysregulation, or increased oncotic pressure [9].

Poor control of factors influencing CT could be responsible for the conflicting results in the literature: axial length and refractive error, diurnal variation of CT, age (there is a decreased of subfoveal CT of approximately 3 microns per year of age), dehydration, and other systemic disease, that can affect choroidal vasculature like systemic hypertension [12, 19, 25–27].

Subfoveal CT was greater in diabetic patients without diabetic retinopathy when compared with nondiabetic patients. Microalbuminuria was associated with an increase in subfoveal and temporal choroidal thickness in diabetic patients without diabetic retinopathy when compared with normoalbuminuric patients and nondiabetic patients.

This study suggests that choroidal changes are present in type 2 diabetic patients even before the clinical development of retinopathy.

Larger prospective clinical studies are needed for more complete knowledge about the role of diabetic choroidopathy both in early pathogenesis of DR and in the disease progression. It remains unclear, though, whether diabetic choroidopathy is a predicting, modulating, or causative factor of DR [12]. OCT angiography (OCT-A) has been performed as a useful tool for evaluating retinal vascular abnormalities. Since it allows visualization of the cariocapillary vessels and assess perfusion densities, OCT-A studies are needed to help clarify changes in the choroidal vessels [28].

Our study is not devoid of limitations. First of all, hydration status and circadian variations were not taken in account; secondly, although OCT measurements were always performed by the same experienced examiner, the measurement was manual and susceptible to variations in reproducibility; lastly, even though the blood tests were analyzed, the group of healthy patients could have some pathology not yet diagnosed.

## Figures and Tables

**Table 1:** Diabetic patients without diabetic retinopathy vs. nondiabetic patients.

	Diabetic group (normoalbuminuria or microalbuminuria) (*n* = 110)	Nondiabetic group (*n* = 30)	*p*
Age (years)	66.67 ± 9.49 (44–86)	63.88 ± 8.72 (51–81)	0.338
Gender (male)	60.00%	53.33%	0.212
Mean time of diagnosis (years)	12.34 ± 8.46	—	—
CT (*μ*m)			
Subfoveal	251.08 ± 69.31	246.03 ± 59.41	**0.006**
Nasal	212.36 ± 70.99	210.33 ± 68.11	0.113
Temporal	243.91 ± 63.48	241.87 ± 62.44	0.239

**Table 2:** Diabetic patients without diabetic retinopathy: microalbuminuria vs. normoalbuminuria

	Diabetic patients with normoalbuminuria *n* = 46	Diabetic patients with microalbuminuria *n* = 64	*p*
Age (years)	66.50 ± 10.01 (44–81)	69.6 ± 9.25 (48–86)	0.349
Gender (male)	60.87%	60.94%	0.199
Mean time of diagnosis (years)	11.94 ± 7.65	12.63 ± 8.56	0.333
CT (*μ*m)			
Subfoveal	247.88 ± 81.144	252.53 ± 64.046	**0.046**
Nasal	205.75 ± 82.906	215.36 ± 65.552	0.219
Temporal	241.29 ± 82.822	245.09 ± 53.397	**0.005**

**Table 3:** Diabetic patients without diabetic retinopathy and microalbuminuria vs. non diabetic patients.

	Diabetic patients with microalbuminuria *n* = 64	Nondiabetic group *n* = 30	*p*
Age (years)	69.6 ± 9.25 (48–86)	63.88 ± 8.72 (51–81)	0.487
Gender (male)	60.94%	53.33%	0.292
Mean time of diagnosis (years)	12.63 ± 8.56	—	—
CT (*μ*m)			
Subfoveal	252.53 ± 64.046	246.03 ± 59.41	**0.049**
Nasal	215.36 ± 65.552	210.33 ± 68.11	0.219
Temporal	245.09 ± 53.397	241.87 ± 62.44	**0.005**

## Data Availability

The data used to support the findings of this study are available from the corresponding author upon request.
